# Diagnostic Performance of Cardiac CT and Transthoracic Echocardiography in Congenital Heart Disease: A Surgical Correlation Study

**DOI:** 10.3390/diagnostics16020259

**Published:** 2026-01-14

**Authors:** Shiraslan Bakhshaliyev, Ergin Arslanoğlu, Heydar Huseynov, Damla İnce, Bahruz Aliyev, Fatih Yiğit, Bulent Polat, Cenap Zeybek

**Affiliations:** 1 Pediatric Cardiovascular Surgery Department, Liv Bona Dea Hospital, Baku 1060, Azerbaijan; 2 Pediatric Cardiovascular Surgery Department, Atlas University Hospital, 34203 Istanbul, Turkey; 3 Radiology Department, Atlas University Hospital, 34203 Istanbul, Turkey; 4 Pediatric Cardiology Department, Atlas University Hospital, 34203 Istanbul, Turkey; 5 Pediatric Cardiology Department, Liv Bona Dea Hospital, Baku 1060, Azerbaijan; 6 Cardiovascular Surgery Department, Kosuyolu High Specialitiason and Research Hospital, University of Health Sciences, 34668 Istanbul, Turkey

**Keywords:** congenital heart disease, multislice computed tomography, transthoracic echocardiography

## Abstract

**Background**: This study evaluated the diagnostic efficacy of transthoracic echocardiography (TTE) and 128-slice multislice computed tomography (MSCT) angiography in congenital heart disease (CHD). **Methods**: Between January 2018 and August 2022, 50 patients diagnosed with CHD underwent both TTE and ECG-gated 128-MSCT. The imaging findings were compared with intraoperative observations, categorizing pathologies into cardiac, heart–great vessel, and great vessel malformations. **Results**: The median age of the patients was 0.45 months, and the median weight was 5 kg. Echocardiography showed a sensitivity of 89.8% and specificity of 99.12%, with an overall accuracy of 98%. MSCT had a sensitivity of 87.9%, specificity of 98.95%, and accuracy of 97.62%. There was no significant difference in diagnostic accuracy between the two modalities (χ^2^ = 31.796, *p* = 0.215), with substantial agreement (kappa = 0.901). For surgically confirmed cardiac malformations (*n* = 69), echocardiography had a 100% sensitivity, whereas MSCT had an 88.41% sensitivity (χ^2^ = 20.633, *p* = 0.039), with high concordance (kappa = 0.931). For heart–great vessel connection anomalies (*n* = 27), both modalities had an 81.48% sensitivity (χ^2^ = 14.115, *p* = 0.481), with substantial agreement (kappa = 0.887). For great vessel malformations (*n* = 61), the echocardiography and MSCT sensitivities were 81.97% and 88.52%, respectively, with no significant difference in performance (χ^2^ = 30.303, *p* = 0.063), indicating substantial concordance (kappa = 0.863). **Conclusions**: Both TTE and MSCT are highly accurate for CHD diagnosis, each with unique advantages. Their complementary use, especially where one modality is limited, enables a more comprehensive assessment, supporting clinical decision-making and surgical planning.

## 1. Background

Congenital heart disease (CHD) is the most common birth defect, affecting approximately 8–10 children per 1000 live births. Over the past few decades, advancements in diagnostic techniques and surgical interventions have significantly improved survival rates, with nearly 90% of CHD patients now reaching adulthood. The management and follow-up of this heterogeneous group, characterized by a wide morphological spectrum, rely heavily on accurate diagnostic tools. Surgical intervention during infancy or even the neonatal period can substantially improve long-term outcomes for some complex cardiac malformations. Therefore, the early diagnosis and confirmation of CHD, alongside the creation of appropriate surgical treatment opportunities, are critically important [1].

Transthoracic echocardiography (TTE) and cardiac catheterization are fundamental tools for diagnosing complex congenital heart defects. TTE, particularly when combined with color Doppler, provides detailed visualization of intracardiac anatomy, including hemodynamic evaluation. However, TTE is operator dependent and has limitations in imaging extracardiac structures such as pulmonary arteries, pulmonary veins, the aortic arch, major vessels, and airway abnormalities [2].

Recent advancements in cardiac CT technology have contributed to the expanded use of computed tomography (CT) in CHD patients by mitigating issues such as cardiac motion artifacts and radiation exposure. Multislice spiral computed tomography (MSCT) is a robust technique capable of providing high-resolution visualization of the morphology of complex CHDs [3]. Features such as flexible ECG-synchronized scanning, low radiation doses, high scanning speeds, and expanded anatomical coverage are critical for improving image quality while minimizing patient risk [4].

This study aims to evaluate the diagnostic performance of computed tomography angiography (CTA) compared to echocardiography and its potential complementary role in the diagnosis of congenital heart diseases. By comparing the diagnostic findings with surgical outcomes, this study sought to determine the complementary role of CTA in enhancing diagnostic accuracy and supporting clinical decision-making.

## 2. Methods

This retrospective study was approved by the Istanbul Atlas University Ethics Committee (Approval No: E-22686390-050.99-21527, Date: 8 November 2022) and was conducted in accordance with the principles of the Declaration of Helsinki. Written informed consent was obtained from the parents or legal guardians of all pediatric patients.

Between January 2018 and August 2022, 50 patients diagnosed with CHD in our clinic underwent TTE followed by electrocardiogram (ECG)-gated 128-MSCT. Only patients who underwent both transthoracic echocardiography (TTE) and ECG-gated multislice computed tomography (MSCT) followed by surgical correction between January 2018 and August 2022 were included, allowing direct correlation with intraoperative findings. This approach may lead to selection bias toward more complex congenital heart lesions. The patients were retrospectively evaluated, and the imaging findings were compared with intraoperative observations. Pathologies were categorized into three groups:

1. Cardiac malformations;
2. Heart–great vessel connection anomalies;
3. Great vessel malformations.

### 2.1. Echocardiography Protocol

Echocardiographic evaluations were performed via a GE Vivid S5 cardiac ultrasound system (General Electric Vingmed, Horten, Norway) equipped with a 3 MHz or 6 MHz transducer. Cardiac functions were assessed via various imaging modalities, including biplanar 2D echocardiography, 2D color flow Doppler imaging, and conventional B-mode 2D echocardiography. The procedures for obtaining optimal cardiac acoustic windows were meticulously followed. Echocardiographic imaging typically commenced with a subcostal acoustic window, and major cardiovascular structures were evaluated according to the Van Praagh segmental analysis. All echocardiographic studies were performed by two senior pediatric cardiologists with over ten years of experience in congenital heart imaging. All echocardiographic and MSCT images were retrieved from the institutional PACS and re-evaluated by the same experienced operators to ensure consistency across interpretations.

### 2.2. MSCT Protocol

The ECG-gated thoracic angiography protocol was specifically designed to provide detailed visualization of thoracic and cardiac structures in pediatric patients. MSCT scans were performed via a dual-source 128-slice CT scanner equipped with two X-ray tubes and dual detector arrays (Somatom Definition Flash^®^, Siemens Healthcare, Forchheim, Germany). Intravenous access was established on the basis of the patient’s weight and age. A contrast agent, Opaxol (Iohexol) (Opakim Medical Products, Istanbul, Türkiye) 300 mg/mL, was administered intravenously at 1 cc per kilogram of body weight, followed by a 20 cc bolus of 0.9% NaCl saline solution. Imaging was conducted in helical mode with ECG gating to minimize motion artifacts during both the systolic and diastolic phases. The slice thickness was set to 1.25 mm, with a reconstruction thickness of 0.625 mm. The tube voltage (kVp) and current (mAs) values were optimized according to the patient’s weight and age via automatic dose modulation.

The scan field extended from the upper border of the thorax to the lower border of the diaphragm. Patients were placed in a supine position with their arms extended above their heads. Axial, coronal, and sagittal images were acquired to evaluate the lung parenchyma and vascular structures. Late-phase imaging was performed after the systemic circulation of the contrast agent to assess contrast distribution and pathological formations. The acquired images were processed via multiplanar reconstruction (MPR) and three-dimensional volumetric reconstruction techniques, enabling detailed evaluation of the lung parenchyma, pulmonary vessels, and mediastinal structures (Figure S1). Late-phase imaging was specifically used to assess contrast dynamics and pathological findings [5].

### 2.3. Statistical Design

The diagnostic accuracy and kappa values of TTE and MSCT were assessed on the basis of surgical findings. Data analysis was conducted via IBM SPSS Statistics Version 27 (Armonk, NY, USA). Categorical variables are expressed as frequencies and percentages, whereas continuous variables with nonnormal distributions are reported as medians (25–75th percentiles). The diagnostic accuracy of the two methods was compared via McNemar’s test. Diagnostic agreement was assessed via kappa statistics (κ), which measures agreement beyond chance; κ values were interpreted as <0.20 slight, 0.21–0.40 fair, 0.41–0.60 moderate, 0.61–0.80 substantial, and 0.81–1.00 almost perfect agreement. The chi-square (χ^2^) test was used for comparing categorical variables between groups where appropriate. The area under the curve (AUC) for both methods was evaluated via receiver operating characteristic (ROC) analysis. A *p* value of <0.05 was considered to indicate statistical significance. Intraoperative surgical findings were considered the gold standard reference for calculating diagnostic test performance metrics (sensitivity, specificity, positive predictive value [PPV], negative predictive value [NPV], and accuracy).

## 3. Results

The study cohort comprised 50 patients, 46% of whom were male. The median age was 0.45 months (range: 0.1–10 months), and the median weight was 5 kg (range: 4–8 kg). The distributions of cardiac malformations, heart–great vessel connection anomalies, and great vessel malformations are detailed in Table 1.

Table 2 presents all identified malformations, including true positives, false positives, and false negatives, as determined by TTE and MSCT compared with surgical diagnoses. In the assessment of cardiac malformations via TTE, there were false positive results in 5 patients for ASD, 1 for left ventricular outflow tract obstruction (LVOTO), and 1 for mitral valve disease. MSCT evaluations yielded a single false positive for mitral valve disease. The false negative findings included 6 cases of ASD and 2 cases of mitral valve disease.

In the group with combined cardiac and vascular anomalies, neither TTE nor MSCT produced false positive diagnoses. However, TTE failed to detect coronary artery anomalies in 5 patients (false negatives). MSCT missed 2 cases of transposition of the great arteries (TGAs) and 2 cases of pulmonary valve pathology.

Within the great vessel malformation category, TTE yielded false positives in 1 patient for aortic coarctation and 2 patients for aortic arch hypoplasia. MSCT identified false positives in 1 case of aortic coarctation, 3 cases of aortic arch hypoplasia, 1 case of pulmonary artery (PA) stenosis, 4 cases of major aortopulmonary collateral artery (MAPCA), 1 case of pulmonary venous drainage anomaly (PVDA), and 1 case of patent ductus arteriosus (PDA). TTE missed diagnoses in 2 patients with aortic arch hypoplasia: 1 with PA stenosis, 2 with PVDA, 2 with PDA, and 4 with persistent left superior vena cava (PLSVC). MSCT failed to detect 1 case of aortic arch hypoplasia, 1 case of PVDA, and 3 cases of PDA. Among the atrial septal defects (ASDs), all overdiagnosed cases by echocardiography were secundum-type ASDs, whereas the underdiagnosed lesions by CT involved superior sinus venosus ASDs associated with partial anomalous pulmonary venous drainage. The two CT-missed TGA diagnoses corresponded to complex variants (Taussig–Bing anomaly and DORV with anterior aorta) where segmental analysis was incomplete.

Comparisons between TTE, MSCT, and surgical diagnoses for cardiac malformations are detailed in Table 3 and Table 4.

TTE identified 141 true positives, 1131 true negatives, 11 false positives, and 16 false negatives, resulting in a sensitivity of 89.80% and a specificity of 99.12%. The positive predictive value (PPV) was 93.38%, the negative predictive value (NPV) was 98.61%, and the overall diagnostic accuracy was 98%. The area under the curve (AUC) for TTE was 0.944, which slightly surpassed that of MSCT (0.931).

MSCT detected 137 true positives, 1131 true negatives, 12 false positives, and 20 false negatives, yielding a sensitivity of 87.9%, specificity of 98.95%, PPV of 92%, NPV of 98.23%, and accuracy rate of 97.62%. The comparison between TTE and MSCT revealed no significant difference in diagnostic accuracy (χ^2^ = 31.796, *p* = 0.215), with substantial agreement between the two modalities (kappa = 0.901).

For surgically confirmed cardiac malformations (*n* = 69), the TTE and MSCT sensitivities were 100% and 88.41%, respectively (χ^2^ = 20.633, *p* = 0.039), indicating high concordance (kappa = 0.931). For heart–great vessel connection anomalies (*n* = 27), both TTE and MSCT demonstrated sensitivities of 81.48% (χ^2^ = 14.115, *p* = 0.481), with substantial agreement (kappa = 0.887). With respect to great vessel malformations (*n* = 61), the TTE and MSCT sensitivities were 81.97% and 88.52%, respectively, with no significant difference in diagnostic performance (χ^2^ = 30.303, *p* = 0.063) or substantial concordance (kappa = 0.863).

When TTE and MSCT findings were combined—considering a diagnosis accurate if detected by either modality—the overall sensitivity increased to 96.8%, specificity to 98.1%, and accuracy to 98.5%, emphasizing their complementary diagnostic value.

## 4. Discussion

It is important to note that our study was not designed as a prospective trial evaluating the incremental value of adding MSCT to TTE based on clinical endpoints such as changes in surgical strategy. Instead, our study aimed to compare the individual diagnostic performances of TTE and MSCT in a cohort of patients who underwent both imaging modalities, and to highlight their complementary roles. This approach helps in understanding the unique strengths and weaknesses of each modality. These findings indicate that both modalities provide high diagnostic accuracy in detecting anatomically confirmed structures. However, each method’s distinct advantages and limitations may influence its clinical application in specific scenarios.

TTE is a fundamental diagnostic tool for CHD, offering clear identification of defect anatomy through segmental evaluation. In cases of simple cardiac defects such as ASD, VSD, and PDA, TTE often supplies sufficient preoperative information without necessitating additional diagnostic methods. In this study, TTE demonstrated 100% sensitivity in detecting intracardiac malformations, which aligns with the literature. For example, a study by Öztürk et al. reported that TTE has high sensitivity and specificity in identifying intracardiac anomalies. The superiority of TTE in evaluating cardiac chambers and valve structures is well documented, as it provides dynamic, real-time imaging that details the intricacies of blood flow [6]. Its noninvasive nature, bedside availability, and absence of radiation exposure make it a repeatable and essential tool for preoperative, intraoperative, and postoperative monitoring of congenital heart surgery patients. However, TTE limitations include operator dependency, subjective assessment, and challenges in visualizing extracardiac vascular structures owing to echogenicity and image quality constraints.

Previous studies have demonstrated the success of TTE in detecting CHD. Öztürk et al. reported that the sensitivity of TTE was 94.3%, the specificity was 99.4%, and the diagnostic accuracy was 98.4%. Another study reported that the sensitivity and specificity of TTE were 90.6% and 99.8%, respectively [6,7,8]. In our study, TTE exhibited a sensitivity of 89.8% and a diagnostic specificity of 99.12%. The positive predictive value was 93.38%, and the negative predictive value was 98.61%, with an overall diagnostic accuracy of 98%. These findings are consistent with the literature.

MSCT has become a critical imaging modality in the diagnosis and management of CHD and is particularly valuable in surgical planning and complex interventions requiring detailed anatomical visualization. Three-dimensional reconstructions are especially beneficial in planning surgeries for complex vascular anomalies such as aortopulmonary collaterals [9]. Modern CT devices complete imaging within seconds, significantly reducing the need for sedation or general anesthesia in pediatric patients [10]. Compared with traditional catheter-based angiography, CT is noninvasive, associated with lower complication rates, and exposes patients to less radiation [11,12].

Nonetheless, MSCT has several limitations. Despite advancements in dose reduction technologies, radiation exposure remains a concern, particularly in pediatric populations with longer life expectancies. Techniques such as prospective ECG synchronization and iterative reconstruction have mitigated this risk but have not eliminated it [13]. Iodine-based contrast agents used in CT carry a risk of nephrotoxicity, especially in neonates and patients with compromised renal function. Additionally, MSCT does not provide functional data such as ventricular ejection fraction or valve insufficiencies, necessitating supplementary imaging modalities for comprehensive assessment [14].

In this study, the sensitivity of TTE for detecting great vessel malformations was 81.97%, whereas the sensitivity of MSCT was 88.52%, indicating the superiority of MSCT in visualizing extracardiac vascular structures. The literature suggests that MSCT offers greater diagnostic accuracy, particularly in conditions such as pulmonary artery stenosis, PDA, and major vascular anomalies [15,16]. Our findings corroborate that MSCT outperforms ECHO in cases such as PDA and aortic arch hypoplasia. However, the difference between the two modalities was not statistically significant (*p* = 0.063).

The study also revealed that TTE yielded false positive results in some ASD and mitral valve disease cases, whereas MSCT led to false negative outcomes in certain instances. This aligns with the literature highlighting the operator dependency and image quality limitations of TTE, which can hinder the detection of small or complex lesions. Conversely, MSCT’s provision of static images without hemodynamic assessment may result in missed diagnoses of dynamic cardiac lesions.

Recent technological advancements have addressed some of the disadvantages of MSCT. Integration with 3D printing and augmented reality is revolutionizing surgical planning. Innovations such as photon-counting detectors and artificial intelligence-based dose optimization are expected to further reduce radiation exposure while enhancing image quality. Emerging technologies such as dynamic CT perfusion imaging are expanding the functional assessment capabilities of MSCT, positioning it as a competitor to magnetic resonance imaging [17].

TTE and MSCT are complementary modalities for CHD diagnosis. The literature indicates that TTE is more effective for detecting intracardiac anomalies, whereas MSCT excels in evaluating complex vascular structures. Our study revealed high concordance between the two methods (Kappa = 0.901).

## 5. Limitations

This study has several limitations, including a limited sample size and its retrospective, single-center design. Additionally, TTE results are operator-dependent, potentially affecting accuracy, especially among less experienced practitioners. Similarly, MSCT interpretations by individuals with limited experience may lead to diagnostic errors. A key limitation of this study is that it did not include a TTE-only control group or assess clinical endpoints such as surgical strategy changes, which prevents direct measurement of MSCT’s incremental diagnostic value. Future prospective studies are warranted to address this gap. Additionally, MSCT interpretations were informed by prior TTE results. While this reflects real-world clinical practice, the lack of a blinded re-evaluation may have introduced interpretation bias. Additionally, MSCT interpretations were informed by prior TTE results, introducing possible incorporation bias.

## 6. Conclusions

TTE and MSCT are highly accurate diagnostic tools for CHD. Each offers unique advantages, and their complementary use can allow for a more comprehensive and precise assessment, particularly in situations where one modality has limitations. Further research involving larger patient cohorts is essential to delineate the specific applications and potential incremental value of these modalities. These findings provide clinicians with valuable insights to support clinical decision-making and potentially achieve improved surgical outcomes.

## Figures and Tables

**Table 1:** Baseline Characteristics.

		*n* (%)/Median (IQR)
GENDER	female	27 (54%)
	male	23 (46%)
weight (/kg)		5 (4–8)
age (/month)		0.45 (0.1–10)
Cardiac malformations	−	11 (22%)
	+	39 (78%)
Heart–great vessel connection anomalies	−	34 (68%)
	+	16 (32%)
Great vessel malformations	−	2 (4%)
	+	48 (96%)

IQR: Interquartile Range.

**Table 2:** Distributions of diagnoses by TTE, MSCT and surgery.

Malformation Type		TTE			MSCT			Malformation
		TP	FP	FN	TP	FP	FN	
Cardiac malformations	VSD	30	0	0	30	0	0	30
	ASD	24	5	0	18	0	6	24
	Single Atrium	1	0	0	1	0	0	1
	Single Ventricle	2	0	0	2	0	0	2
	RVOTO	3	0	0	3	0	0	3
	LVOTO	2	1	0	2	0	0	2
	Mitral Valve Deformity	3	1	0	1	1	2	3
	Tricuspit Valve Deformity	1	0	0	1	0	0	1
	Cor triatrium sinistrum	1	0	0	1	0	0	1
	AVSD	2	0	0	2	0	0	2
Heart–great vessel connection anomalies	TGA	13	0	0	11	0	2	13
	DORV	1	0	0	1	0	0	1
	Aorta valve	0	0	0	0	0	0	0
	Pulmonary valve	7	0	0	5	0	2	7
	Coronary artery	1	0	5	6	0	0	6
Great vessel malformations	Coarctation of aorta	10	1	0	10	1	0	10
	Arcus hipo	4	2	2	5	3	1	6
	IAA	1	0	0	1	0	0	1
	PA dilatation	1	0	0	1	0	0	1
	PA stenosis	5	0	1	6	1	0	6
	MAPCA	1	0	0	1	4	0	1
	PVDA	1	0	2	2	1	1	3
	PDA	21	0	2	20	1	3	23
	PLSVC	3	0	4	5	0	2	7
	Double Arc	1	0	0	1	0	0	1
	AP window	2	0	0	2	0	0	2

TTE: Transthoracic Echocardiography; MSCT: Multislice Computed Tomography; TP: True Positive; FP: False Positive; FN: False Negative; VSD: Ventricular Septal Defect; ASD: Atrial Septal Defect; RVOTO: Right Ventricular Outflow Tract Obstruction; LVOTO: Left Ventricular Outflow Tract Obstruction; AVSD: Atrioventricular Septal Defect; TGA: Transposition of the Great Arteries; DORV: Double Outlet Right Ventricle; IAA: Interrupted Aortic Arch; PA: Pulmonary Artery; MAPCA: Major Aortopulmonary Collateral Artery; PVDA: Pulmonary Venous Drainage Anomaly; PDA: Patent Ductus Arteriosus; PLSVC: Persistent Left Superior Vena Cava; AP window: Aortopulmonary Window.

**Table 3:** Distribution of Diagnostic Variables in TTE and MSCT Modalities.

**All Patients**							
Method	sensitivity	** specificity **	PPV	NPV	AUC	Accuracy	Kappa
TTE	0.898	0.9912	0.9338	0.9861	0.944	0.98	0.901
MSCT	0.879	0.9895	0.92	0.9823	0.931	0.9762	
** Cardiac malformations **							
Method	sensitivity	** specificity **	PPV	NPV	AUC	Accuracy	Kappa
TTE	1	0.971	0.8961	1	0.991	0.982	0.931
MSCT	0.8841	0.9988	0.9839	0.9836	0.896	0.982	
** Heart–great vessel connection anomalies **							
Method	sensitivity	** specificity **	PPV	NPV	AUC	Accuracy	Kappa
TTE	0.8148	1	1	0.9772	0.907	0.98	0.887
MSCT	0.8148	1	1	0.9772	0.907	0.98	
** Great vessel malformations **							
Method	sensitivity	** specificity **	PPV	NPV	AUC	Accuracy	Kappa
TTE	0.8197	0.9939	0.9434	0.9779	0.907	0.954	0.863
MSCT	0.8852	0.9786	0.8308	0.9856	0.884	0.9485	

TTE: Transthoracic Echocardiography; MSCT: Multislice Computed Tomography; PPV: Positive Predictive Value; NPV: Negative Predictive Value; AUC: Area Under the Curve. Specificity and Kappa values are presented.

**Table 4:** Comparison of the Diagnostic Power of TTE and MSCT Modalities.

**All Patients**		**Operation Diagnosis**			
		−	**+**		
		**Count**	**Count**	**Chi-Square**	***p***
TTE	−	1131	16	31,796	0.215
	+	11	141		
MSCT	−	1131	20		
	+	12	137		
** Cardiac Malformations **		**Operation Diagnosis**			
		**−**	**+**		
		**Count**	**Count**	**Chi-Square**	***p***
TTE	−	422	0	20,633	0.039
	+	8	69		
MSCT	−	430	8		
	+	1	61		
** Heart–Great Vessel Connection Anomalies **		**Operation Diagnosis**			
		**−**	**+**		
		**Count**	**Count**	**Chi-Square**	***p***
TTE	−	223	5	14,115	0.481
	+	0	22		
MSCT	−	223	5		
	+	0	22		
** Great Vessel Malformations **		**Operation Diagnosis**			
		**−**	**+**		
		**Count**	**Count**	**Chi-Square**	***p***
TTE	−	486	11	30,303	0.063
	+	3	50		
MSCT	−	478	7		
	+	11	54		

TTE: Transthoracic Echocardiography; MSCT: Multislice Computed Tomography.

## Data Availability

The data presented in this study are available from the corresponding author upon reasonable request. Public sharing of the data is restricted due to ethical approval conditions and the need to protect patient confidentiality.

## Supplementary Materials

The following supporting information can be downloaded at: https://www.mdpi.com/article/10.3390/diagnostics16020259/s1, Figure S1: Representative CT Images Demonstrating Discrepant or Complementary Findings.

## References

[1] Glöckler M., Halbfaß J., Koch A., Dittrich S., Achenbach S., Rüffer A., Ihlenburg S., Cesnjevar R., May M., Uder M. (2014). Preoperative assessment of the aortic arch in children younger than 1 year with congenital heart disease: Utility of low-dose high-pitch dual-source computed tomography. A single-center, retrospective analysis of 62 cases. Eur. J. Cardiothorac. Surg..

[2] Mertens L., Friedberg M.K. (2009). The gold standard for noninvasive imaging in congenital heart disease: Echocardiography. Curr. Opin. Cardiol..

[3] Cheng Z., Wang X., Duan Y., Wu L., Wu D., Chao B., Liu C., Xu Z., Li H., Liang F. (2010). Low-dose prospective ECG-triggering dual-source CT angiography in infants and children with complex congenital heart disease: First experience. Eur. Radiol..

[4] Koplay M., Kizilca O., Cimen D., Sivri M., Erdogan H., Guvenc O., Oc M., Oran B. (2016). Prospective ECG-gated high-pitch dual-source cardiac CT angiography in the diagnosis of congenital cardiovascular abnormalities: Radiation dose and diagnostic efficacy in a pediatric population. Diagn. Interv. Imaging.

[5] Cohen J., Asrani P., Lee S., Frush D., Han B.K., Chelliah A., Farooqi K.M. (2022). Cardiovascular computed tomography in pediatric congenital heart disease: A state-of-the-art review. J. Cardiovasc. Comput. Tomogr..

[6] Öztürk E., Tanıdır İ.C., Kamalı H., Ayyıldız P., Topel C., Onan I.S., Türkvatan A., Haydin S., Güzeltaş A. (2021). Comparison of echocardiography and 320-row multidetector computed tomography for the diagnosis of congenital heart disease in children. Rev. Port. Cardiol. (Engl. Ed.).

[7] Bu G., Miao Y., Bin J., Deng S., Liu T., Jiang H., Chen W. (2016). Comparison of 128-slice low-dose prospective ECG-gated CT scanning and transthoracic echocardiography for the diagnosis of complex congenital heart disease. PLoS ONE.

[8] Li A., Peng Z., Zhang C. (2017). Comparison of echocardiography and 64-multislice spiral computed tomography for the diagnosis of pediatric congenital heart disease. Med. Sci. Monit..

[9] Dodge-Khatami J., Adebo D.A. (2021). Evaluation of complex congenital heart disease in infants using low-dose cardiac computed tomography. Int. J. Cardiovasc. Imaging.

[10] Pushparajah K., Duong P., Mathur S., Babu-Narayan S.V. (2019). Cardiovascular MRI and CT in congenital heart disease. Echo Res. Pract..

[11] Shi K., Yang Z.G., Xu H.Y., Zhao S.X., Liu X., Guo Y.K. (2016). Dual-source computed tomography for evaluating pulmonary artery in pediatric patients with cyanotic congenital heart disease: Comparison with transthoracic echocardiography. Eur. J. Radiol..

[12] Al-Mousily F., Shifrin R.Y., Fricker F.J., Feranec N., Quinn N.S., Chandran A. (2011). Use of 320-detector computed tomographic angiography for infants and young children with congenital heart disease. Pediatr. Cardiol..

[13] Siripornpitak S., Pornkul R., Khowsathit P., Layangool T., Promphan W., Pongpanich B. (2013). Cardiac CT angiography in children with congenital heart disease. Eur. J. Radiol..

[14] Küçükdurmaz Z., Karapınar H., Göktekin Ö. (2012). Konjenital kalp hastalıklarında manyetik rezonans görüntüleme ve bilgisayarlı tomografi. Koşuyolu Kalp Derg..

[15] Mei M., Nie J., Yang Z.S., Sun H.W., Wang H., Kang X.M. (2019). Comparison of echocardiography and 64-slice spiral computed tomography in the diagnosis of congenital heart disease in children. J. Cell. Biochem..

[16] Joshi S., Guleria M., Kumar D., Bhatt D., Arora S., Jadon R.S. (2023). Comparison of echocardiography and computed tomography angiography in the evaluation of pulmonary arteries and pulmonary annulus in congenital heart diseases with right ventricular outflow tract obstruction. J. Ind. Acad. Echocardiogr. Cardiovasc. Imaging.

[17] Sachdeva R., Armstrong A.K., Arnaout R., Grosse-Wortmann L., Han B.K., Mertens L., Moore R.A., Olivieri L.J., Parthiban A., Powell A.J. (2024). Novel techniques in imaging congenital heart disease: Advances in echocardiography, cardiac magnetic resonance, cardiac computed tomography, invasive angiography, 3-D visualization. J. Am. Coll. Cardiol..

