# Supplementary material for: Diagnostic Performance of Cardiac CT and Transthoracic Echocardiography in Congenital Heart Disease: A Surgical Correlation Study
Source: Diagnostics (Basel). 2026 Jan 14;16(2):259. doi: 10.3390/diagnostics16020259 (PMC12839741; doi:10.3390/diagnostics16020259)
Supplement: Supplementary file 1 [file diagnostics-16-00259-s001.zip › diagnostics-3904677-supplementary.pdf]

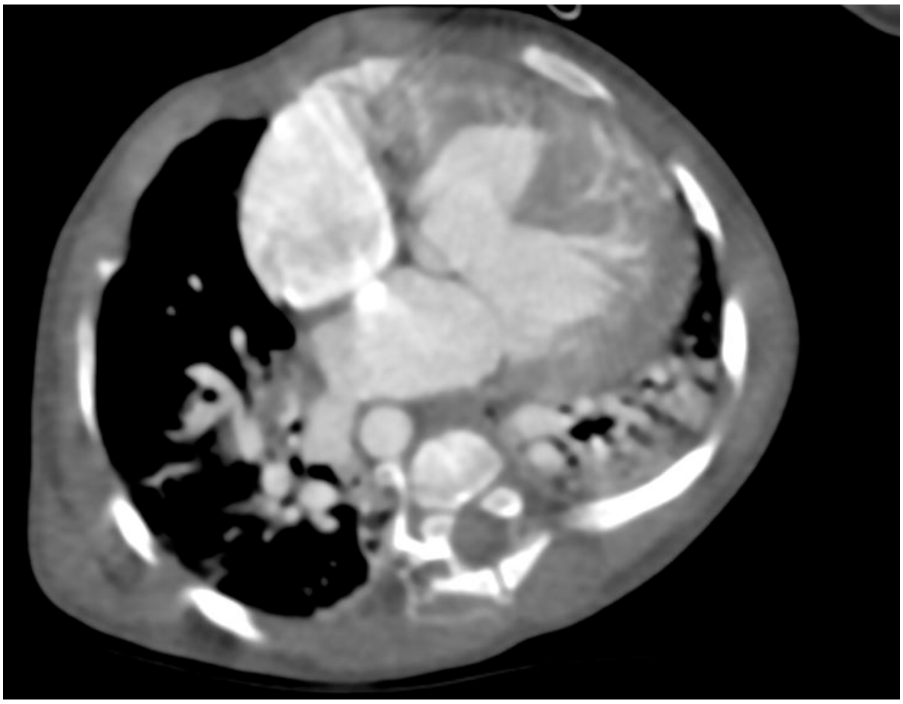

(**A**)

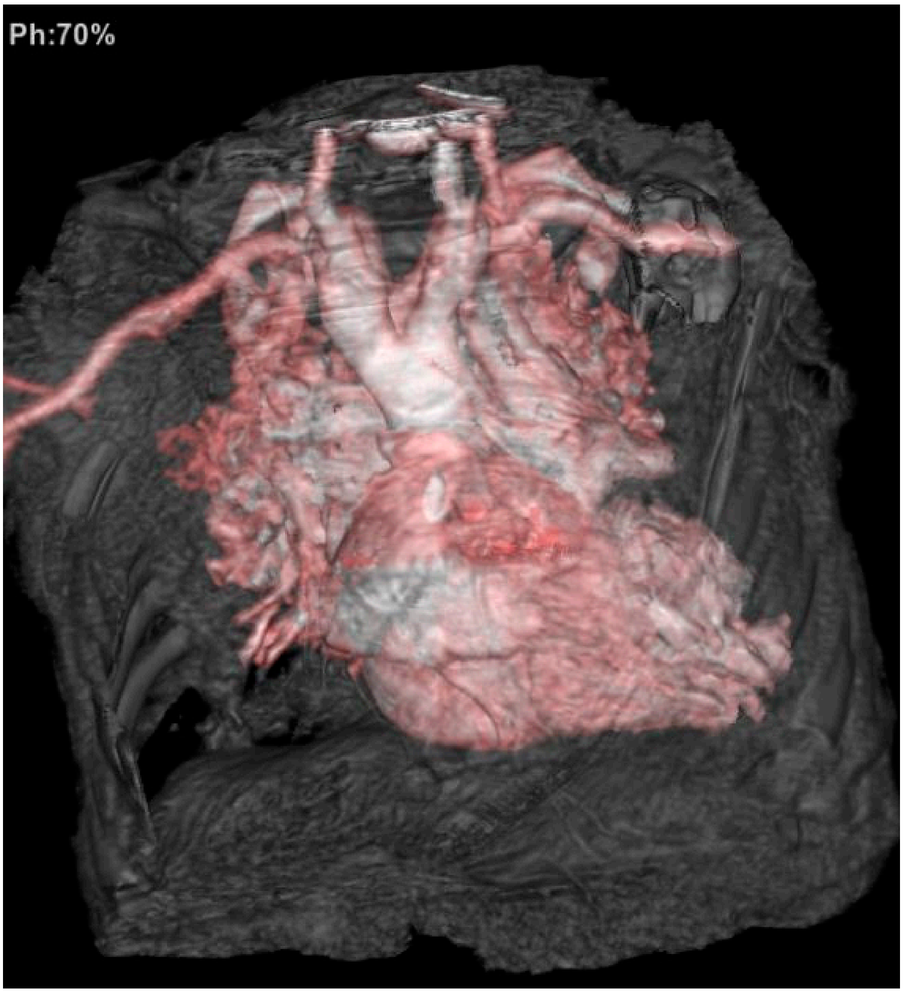

(**B**)

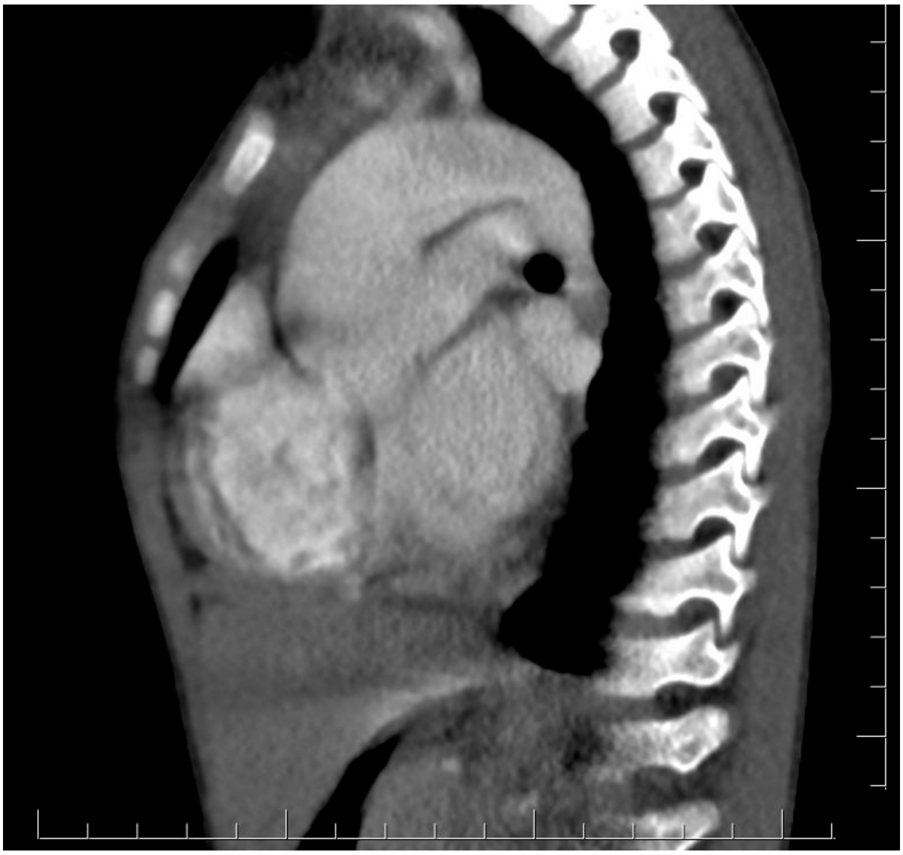

(**C**)

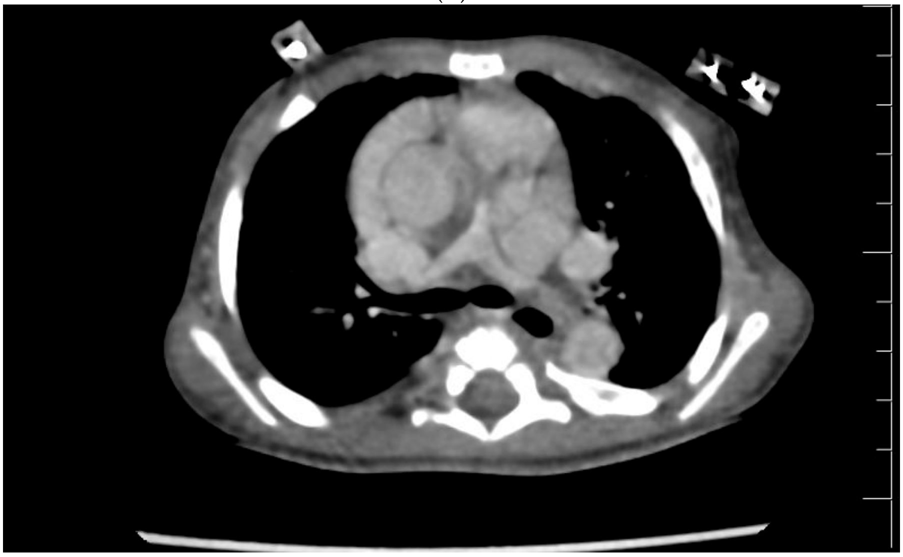

(**D**)

Figure S1. Representative CT Images Demonstrating Discrepant or Complementary Findings. (**A**) Secundum atrial septal defect (ASD) — subtle interatrial septal defect not optimally visualized on CT, but clearly seen on echocardiography. (**B**) Double aortic arch — CT accurately delineates both arches and their spatial orientation, whereas echocardiography suggested vascular compression only. (**C**) Truncus arteriosus type I — single truncal vessel and branch pulmonary arteries are well defined on CT, providing superior

anatomical detail to complement echo findings. (**D**) Ventricular septal defect with pulmonary atresia — CT demonstrates pulmonary artery discontinuity and collateral circulation, not clearly identifiable on echocardiography.
